# Amœbæ in an Abscess of the Jaw

**Published:** 1893-01

**Authors:** 


Amœbæ in an Abscess of the Jaw.—Flexner (*Johns Hopkins Bulletin*, 1892, No. 25, p. 104) has reported the case of a man, sixty-two years old, who, nearly two years before coming under observation, had noticed a small hard lump within the mouth, beneath the gum, at the anterior part of the inferior maxillary bone, just to the right of the middle line. After two months the tumor was removed by an operation. Five months later an ulcer appeared at the site of operation, exposing the bone. A slight discharge set in and continued thereafter. In the course of fourteen months a hard swelling again appeared on the floor of the mouth, gradually increasing and extending beneath the chin to the front of the neck and below the jaw, forming a large and prominent swelling that occupied the entire space included within the arch of the inferior maxillary bone. The width of the mass corresponded with that of the lower jaw. The growth extended backward to the angle of the jaw and downward to the cricoid cartilage. It was firm, indurated, and immovably fixed, and felt like a solid growth. At the most prominent point of the tumor in front, however, tenderness was present and fluctuation could be detected. The teeth of the lower jaw were deficient. On the upper aspect of the transverse portion of the inferior maxilla the bone was denuded in a situation corresponding with the two incisors and the canine tooth of the right side. The exposed bone appeared spongy and was evidently necrotic. It was in part covered with dirty grayish purulent matter and shreds of greenish sloughing tissue. On making firm pressure over the tender and fluctuating area on the anterior aspect of the swelling, a small amount of dirty purulent material was observed to ooze from an opening in the mouth over the necrotic bone. The breath was offensive. There was no glandular involvement. An incision evacuated about two and a half ounces of pus and exposed a large abscess cavity, the walls of which were quite thick. The necrotic bone was thoroughly scraped, the abscess cavity packed with iodoform gauze, and a dressing applied. The man did perfectly well, and at the time of the report bade fair to recover. The pus evacuated was grayish-yellow in color and extremely offensive in odor. It was not thick, but was mixed with a small quantity of blood, and contained a number of flakes and granules of lighter color and more opaque than the other constituents. Microscopic examination disclosed the presence of pus-cells, red blood-cells, bacteria, and amœbæ indistinguishable from the amœbæ dysenteriæ; the last were especially abundant in the flakes. Subsequently, examination of the contents of the drainage-tube employed disclosed the presence of the same organisms. There was no history of diarrhœa.

